# Bisphenol A and Its Emergent Substitutes: State of the Art of the Impact of These Plasticizers on Oxidative Stress and Its Role in Vascular Dysfunction

**DOI:** 10.3390/antiox13121468

**Published:** 2024-11-29

**Authors:** José R. Palacios-Valladares, Yesenia I. Martinez-Jimenez, Vanessa Morillon-Torres, Omar B. Rivera-Maya, Rocio Gómez, Emma S. Calderon-Aranda

**Affiliations:** Department of Toxicology, Center for Research and Advanced Studies of the National Polytechnic Institute, Mexico City 07360, Mexico; ricardo.palacios@cinvestav.mx (J.R.P.-V.); yesenia.martinez@cinvestav.mx (Y.I.M.-J.); vanessa.morillon@cinvestav.mx (V.M.-T.); omar.rivera@cinvestav.mx (O.B.R.-M.)

**Keywords:** cardiovascular diseases, endothelial dysfunction, oxidative stress, oxidant system, antioxidant system, bisphenol A, new-generation bisphenols, toxicity

## Abstract

The “One Health approach” has evidenced the significant impact of xenobiotic exposure to health, and humans are a relevant target for their toxic effects. Bisphenol A (BPA) exerts a ubiquitous exposure source in all ecosystems. Given its endocrine-disrupting and harmful consequences on health, several countries have enforced new regulations to reduce exposure to BPA. Cardiovascular diseases (CVDs) are complex conditions that lead to higher mortality worldwide, where family history, lifestyle, and environmental factors, like BPA exposure, have a remarkable contribution. This chemical compound is the most widely used in plastic and epoxy resin manufacturing and has been associated with effects on human health. Therefore, new-generation bisphenols (NGBs) are replacing BPA use, arguing that they do not harm health. Nonetheless, the knowledge about whether NGBs are secure options is scanty. Although BPA’s effects on several organs and systems have been documented, the role of BPA and NGBs in CVDs has yet to be explored. This review’s goals are focused on the processes of endothelial activation (EA)–endothelial dysfunction (ED), a cornerstone of CVDs development, bisphenols’ (BPs) effects on these processes through oxidant and antioxidant system alteration. Despite the scarce evidence on pro-oxidant effects associated with NGBs, our review demonstrated a comparable harmful effect on BPA. The results from the present review suggest that the biological mechanisms to explain BPs cardiotoxic effects are the oxidant stress ↔ inflammatory response ↔ EA ↔ ED → atherosclerotic plate → coagulation promotion. Other effects contributing to CVD development include altered lipid metabolism, ionic channels, and the activation of different intracellular pathways, which contribute to ED perpetuation in a concerted manner.

## 1. Functions and Dysfunctions of the Cardiovascular System: A General Landscape

**Figure 1:**
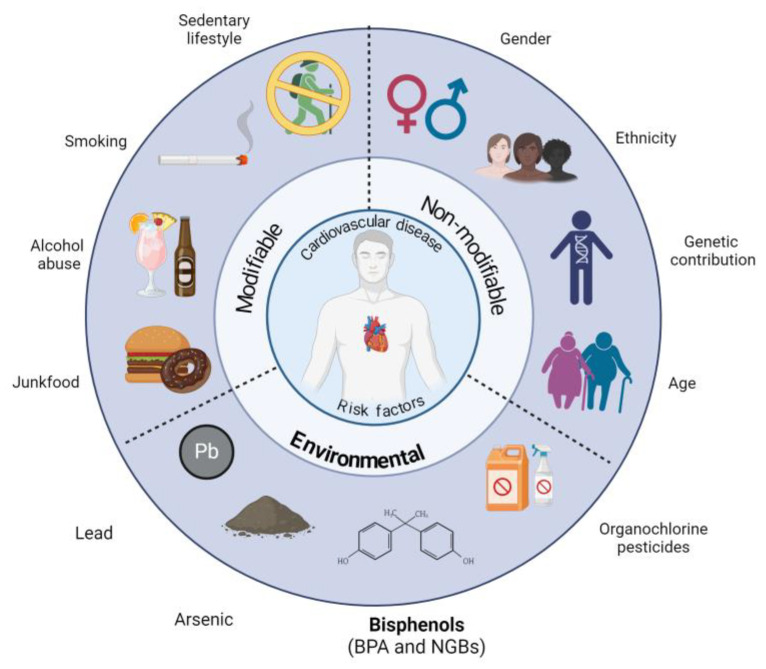
The associated risk factors of cardiovascular diseases. The environmental risk factors appear involved because they are the goal of the present review, particularly bisphenol A and its emergent substitutes (new-generation bisphenols, NGBs). This image was created with BioRender (https://www.biorender.com/ (accessed on 5 September 2024)).

Cardiovascular diseases (CVDs) are complex conditions resulting from the endothelial dysfunction (ED) process. Diet [1], alcohol consumption [2], tobacco use [3], and physical inactivity [4] are well-established modifiable factors contributing to the development and progression of CVDs. In contrast, gender [5], ethnicity [6], genetic predisposition [7], and age [8] are recognized as non-modifiable factors. Furthermore, environmental exposure to pollutants such as lead, arsenic [9], organochlorine pesticides [10], and bisphenol A [11] have been identified as having a central role in the development of CVDs. Hence, environment, genetics, and epigenetics factors, as well as lifestyle, have a critical role in contributing to CVD development, the leading cause of mortality worldwide and one of the causes of disability (Figure 1).

The cardiovascular system (CVS) is lined by the vascular endothelium (VE), which encompasses the heart, arteries, veins, and capillaries; the bloodstream is also contained in the CVS. Under physiological conditions, the VE is selectively permeable, exhibiting in its luminal surface anticoagulant and fibrinolytic properties, preventing intrinsic coagulation cascade activation or platelet adhesion [12]. It also produces autocrine and paracrine molecules and modulates the vascular tone, blood flow, fluid state, and molecules exchanging between the blood and surrounding tissues. Thus, the VE is critical in maintaining homeostasis and hemostasis, aside from controlling arterial tone in response to changes in blood flow through nitric oxide (NO^•−^), an endothelium-derived vasodilator [12]. One of the critical functions of the VE is the balance between oxidants and antioxidants, vasodilators and vasoconstrictors, pro- and anti-inflammatory molecules, and pro- and anti-thrombotic signals, the cornerstone of CVDs [12].

**Figure 2:**
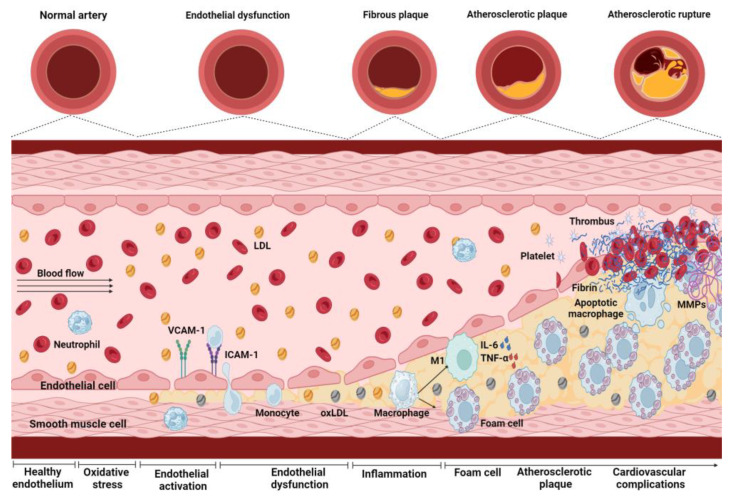
Endothelial dysfunction process and its progression toward atherosclerotic plaque development. Blood vessels are initially present in a healthy endothelium, but endothelial activation (EA) occurs under oxidative stress conditions. EA increases the expression of adhesion molecules, such as vascular cell adhesion molecule 1 (VCAM-1) and intercellular adhesion molecule 1 (ICAM-1), favoring the adhesion and migration of monocytes to the endothelium, contributing, in turn, to the onset of endothelial dysfunction (ED). In ED, the epithelium loses its ability as a selective barrier, increasing vascular permeability and allowing the passage of low-density lipoproteins (LDLs). Once inside the arterial wall, LDL is oxidized, forming ox-LDL. The monocytes migrate to the endothelium and differentiate into macrophages, phagocytizing ox-LDL and becoming foam cells. Simultaneously, M1-type activated macrophages release pro-inflammatory cytokines, such as interleukin (IL) 6 (IL-6) and tumor necrosis factor-alpha (TNF-α), amplifying the inflammatory response at the site of tissue damage. This process is carried out by macrophages, T-cells, natural killer cells, and dendritic cells that contribute to sustained local inflammation and neutrophils that contribute to superficial erosion and fibrous cap rupture. This process contributes to the accumulation of foam cells and the formation of atherosclerotic plaque. Additionally, the activated macrophages secrete matrix metalloproteinases (MMPs), which degrade the extracellular matrix, weakening the atherosclerotic plaque and making it more vulnerable to rupture, increasing the risk of cardiovascular complications. The image was created with BioRender (https://www.biorender.com/ (accessed on 12 September 2024)).

Although atherosclerotic plaque is considered the pathognomonic signature of CVDs, endothelial dysfunction (ED) is the trigger point of such pathologies. ED involves systemic, acute, and chronic changes in endothelial cell function. These changes often occur at sites of disturbed laminar flow, altering shear stress and promoting the atherosclerotic process [13,14]. Yet, endothelial activation (EA), induced by oxidative stress conditions in the VE, is necessary to start the ED process. Hence, EA entails ED, characterized by the loss of VE permeability, causing the trapping of circulating low-density lipoproteins (LDLs) in the subendothelial space, its oxidation (ox-LDL), and accumulation. Concurrently, the VE expresses adhesion molecules such as vascular cell adhesion molecule 1 (VCAM-1), intercellular adhesion molecule 1 (ICAM-1), and E-selectin, concomitant to chemokines, such as monocyte chemotactic protein 1 (MCP-1), which selectively recruit circulating monocytes to the injury site. Monocytes then migrate through the endothelium to the subendothelial layer, differentiate into pro-inflammatory macrophages, and internalize ox-LDL, becoming foam cells, characteristic of early fatty streak lesions [15]. The pro-inflammatory macrophages express cytokines, chemokines, and growth factors. They all induce proliferation and recruitment to the subendothelial layer of neighboring vascular smooth muscle cells that secrete an extracellular matrix that forms a protective fibrous cap around the core of the atheroma [14]. This cap is not a specialized structure but conforms to a typical atherosclerotic fibrous tissue. The atherosclerotic lesion suffers a structural remodeling consisting of a fibrous cap overlying a lipid-rich necrotic core consisting of ox-LDL, cholesterol crystals, and cellular debris. This process is accompanied by matrix remodeling and calcification. The lateral edges of these complex plaques contain pro-inflammatory cells, such as activated macrophages and T-cells, natural killer T-cells, and dendritic cells that contribute to sustained local inflammation. These cells and neutrophils contribute to the structural instability of the atherosclerotic plaque and the proteolytic modification of its extracellular matrix components, with the neutrophils having a predominant role in the propagation of superficial erosion [16]. The lesion could progress to plaque rupture, releasing the highly thrombogenic contents from the necrotic core at the vascular lumen, triggering an atherothrombotic occlusion (Figure 2).

Oxidative stress stimuli are generated by CVD’s concurrent conditions, such as diabetes, metabolic syndrome, obesity, hypercholesterolemia, hypertension, alterations in the renin–angiotensin-II (AngII) system, and inflammation. Such alterations lead to reactive oxygen species (ROS) decreasing the bioavailability of (NO^•−^), a hallmark of vascular EA and a predictive factor of CVD events [17]. Additionally, exposure to xenobiotics, such as bisphenols (BPs), has been related to oxidative stress production, being a possible provider of CVD development as well as other pathologies such as cancer and interstitial lung, kidney, and reproductive dysregulation.

## 2. Bisphenols

BPs are chemicals used to produce polyesters, polyethers [18], polycarbonate plastics, and epoxy resins [19]. Their primary function is to bind, plasticize, reinforce, and serve as a filling material [18]. Bisphenol A (BPA) has been the most widely used globally, with production in 2020 of closer than 7.7 million tons, although it has increased over the years [20]. BPA products include bottles, food and water containers, microwave oven dishes [18], water pipes, the inner linings of food and beverages, receipts [20], toys, and medical and electronic equipment. Given its role in the endocrine system alterations, such compounds have been classified as endocrine-disrupting chemicals (EDCs). BPA is broadly found in the environment and is one of the most common chemical compounds in human and animal matrices [21]. In humans, a dose of 100 mg/kg of deuterated BPA (d6-BPA) administrated for 12 h by dermic via the half-life was 21.4 h to total d6-BPA and 17.6 h to free d6-BPA, and >98% was eliminated after 6 days. [22]. In other living beings, such as Sprague Dawley rats, in 5 min, more than 50% of BPA is conjugated. In this same *in vivo* model, 10 mg/kg of BPA oral via reaches the mean maximum serum concentration (14.7 ng/mL) after 12 min and a half-life of 21.3 h [23].

BPA is a ubiquitously distributed pollutant; its global average estimated daily intake (EDI) is 2.69 ng/kg/day for infants, 60.08 ng/kg/day for children, 42.03 ng/kg/day for pregnant women, and 30.76 ng/kg/day for adults [24]. The tolerable daily intake (TDI) estimates the amount of contaminants in food or water that can be consumed daily over a lifetime without posing a significant health risk. The European Food Safety Authority (EFSA) set the TDI for BPA at 0.2 ng/kg/day [24], a value significantly lower than the reported EDI. Other organizations, such as Health Canada, set a TDI of 25 μg/kg/day [24]. In South Korea, the Ministry of Food and Drug Safety suggested a TDI of 50 μg/kg/day [25]. The TDI value proposed by the EFSA, which is considerably lower than other international standards, highlights the need to consider the risks associated with continuous BPA exposure.

**Figure 3:**
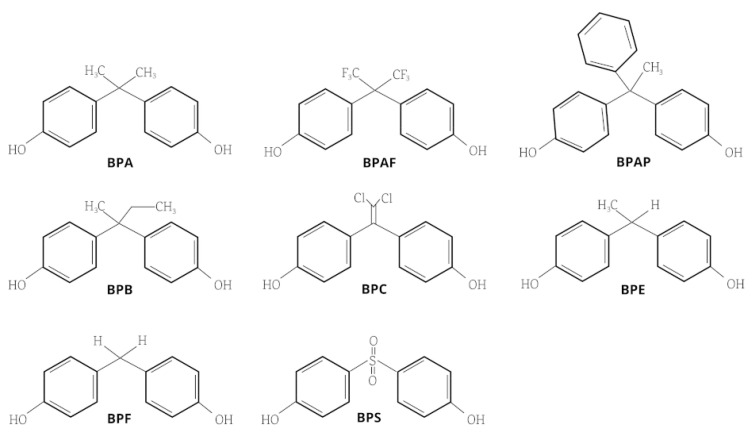
Bisphenols’ chemical structures. Bisphenol A (BPA), bisphenol AF (BPAF), bisphenol AP (BPAP), bisphenol B (BPB), bisphenol C (BPC), bisphenol E (BPE), bisphenol F (BPF), and bisphenol S (BPS).

The “One Health approach” has evidenced the great relevance of xenobiotic exposure and its implications for the health of different ecosystems. These exposures permeate to other life forms, and humans are one of the relevant targets. BPA in the plastics raw materials industry exerts a ubiquitous exposure source in all ecosystems. Consequently, given its endocrine-disrupting effects, several countries have enforced new regulations to reduce their exposure. This regulation has been sustained by the impact of EDCs from natural and anthropogenic sources that interfere with the synthesis, secretion, transport, metabolism, and binding of endogenous hormones, along with their downregulation [26]. The evidence supports that BPA can generate oxidative stress, induce a pro-inflammatory functional profile, and alter lipid metabolism, among other effects involved in ED and consequently in the triggers of CVD development [27]. *Ergo*, its use is being replaced by new-generation bisphenols (NGBs) (Figure 3), arguing that these do not have adverse effects on health and being proposed as “secure alternatives” despite research regarding cardiovascular disorders still being in progress [21,28].

BPA and its emergent chemical substitutes, NGBs, have been associated with damage to different organs and systems, including the CVS [29]. Myocardial infarction, arrhythmias, dilated cardiomyopathy, lipid metabolism dysfunctions, atherosclerosis, and hypertension are well-documented pathologies associated with BPA exposure; *in vitro* models and epidemiological studies have reinforced its impact on CVDs. In this setting, circulating BPA levels have been related to increased intima–media thickness [30,31]. Regarding NGBs’ toxicokinetic properties, scanty evidence is known. However, bisphenol S (BPS) clearance is lower than BPA in piglets and humans, suggesting that it remains in the body longer than BPA, with harmful health consequences [32]. The following sections present evidence of BPA and NGBs’ effects on crucial players of CVD physiopathology, particularly on lipid metabolism, oxidant and antioxidant vascular systems, and other parameters associated with oxidative stress.

## 3. Bisphenol Effects on the Physiopathology of Cardiovascular Disease

### 3.1. Bisphenols and Lipid Metabolism

Bisphenols and their structural homologs have been involved in lipid metabolism [33]. BPA is a potent agonist of the pregnane X receptor (PXR), which induces a proatherogenic effect in humans [34], the accumulation and increase in lipids, cholesterol, and LDL, as well as a decrease in high-density lipoprotein cholesterol (HDL), which also contributes to plaque formation [33]. Human studies have evidenced that alterations in specific genes involved in antiatherogenic functions contribute to clinical manifestations of atherosclerosis, even in middle-aged individuals [35]. Evidence from mice has shown that BPA increases the expression of the genes *Mvd*, *Lss*, *Hmgcr*, and *Sqle*, which are involved in cholesterol biosynthesis [36]. These and other genes affect fatty acid oxidation, synthesis, and absorption [37], increasing lipid droplets, triglyceride levels, and the expression of genes related to lipogenesis, favoring CVD development [38]. In zebrafish, exposure to environmentally relevant concentrations of BPA increases the mRNA expression of genes related to triglyceride transport and decreases the expression of genes related to lipid catabolism [33]. Also, BPA inhibits the expression of ApoE [39] and ApoA [40] in minnow *Gobiocypris rarus*, a relevant finding because of apolipoproteins’ role in triglyceride transport.

Even though few studies have shown that NGBs can alter lipid and cholesterol levels, it was reported that BPB, BPE, BPF, and BPS increase lipid accumulation and alter leptin levels similarly to BPA [41]. BPS and BPF have been associated with greater lipid storage capacity than BPA in pre-adipocytic cells and increase the expression of adipogenic proteins CCAAT/enhancer, binding protein α (C/EBPα), peroxisome proliferator-activated receptor gamma (PPARγ), and acid-binding protein 4 (FABP4) [42]. BPS has been associated with the risk of venous thrombosis because it increases blood lipid and cholesterol levels in cell membranes, morphology, and erythrocyte function [43]. BPAF has been associated with increased lipid content and accelerated maturation of the adipocytes [44].

### 3.2. Bisphenols and the Vascular Oxidant System

**Figure 4:**
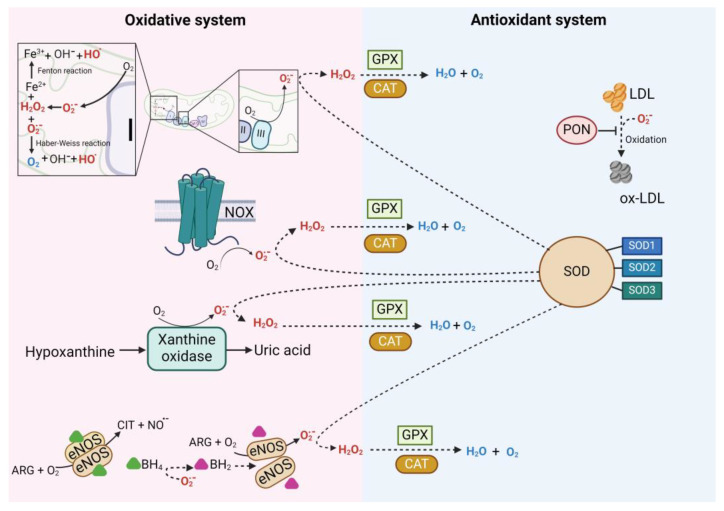
The oxidative and antioxidant system. On the left side are the ROS sources. Complexes I and III of the respiratory chain are the primary sites of reactive oxygen species (ROS) production in mitochondria. Complex I generate O_2_^•−^ on the matrix side, while complex III produces it in the inner mitochondrial membrane and the intermembrane space. The Fenton and Haber–Weiss reactions also occur in the mitochondria, generating ROS and amplifying oxidative stress. The NOX family is a membrane-bound electron-transporting enzyme group that transfers electrons from NADPH to oxygen (O_2_), forming the O_2_^•−^. Xanthine oxidase (XO) catalyzes the oxidation of hypoxanthine to xanthine and xanthine to uric acid, producing O_2_^•−^ and H_2_O_2_. Endothelial nitric oxide synthase (eNOS) is a homodimer dependent on tetrahydrobiopterin (BH_4_), which, under normal conditions, uses oxygen O_2_ and arginine (Arg) to synthesize NO^•−^. However, under oxidative stress, O_2_^•−^ reacts with NO^•−^ to form peroxynitrite (ONOO^−^), which oxidizes the cofactor BH_4_, converting it into dihydrobiopterin (BH_2_), leading to eNOS uncoupling; it produces O_2_^•−^ instead of NO^•−^, increasing oxidative stress. On the right side are the antioxidant enzymes that neutralize ROS and maintain the redox balance. The superoxide dismutase (SOD) system in mammalians includes three isoforms of SOD, namely Cu/Zn-SOD (SOD1), Mn-SOD (SOD2), and extracellular SOD3. SOD1’s primary function is reducing intracellular O_2_^•−^ in the cytosol; SOD2 eliminates O_2_^•−^ from the respiratory chain; SOD3 is the primary antioxidant enzyme secreted into the extracellular space. Catalase (CAT) reduces H_2_O_2_ to H_2_O and O_2_ and is upregulated in response to lipid peroxides. Glutathione peroxidase (GPX) is a selenium-dependent intracellular antioxidant enzyme that inhibits free radical generation from H_2_O_2_ reduction and lipid hydroperoxides to their corresponding alcohols. The Paraoxonase (PON) family, composed of three enzymes (PON1, PON2, PON3), regulates oxidative stress and inflammation, reducing O_2_^•−^ production. The image was created with BioRender (https://www.biorender.com/ (accessed on 25 September 2024)).

The oxidative stress in the vascular endothelium is mediated by superoxide anions (O_2_^•−^) [45], hydroxyl species (HO^−^), and hydrogen peroxide (H_2_O_2_) [46]. The O_2_^•−^ may react with NO^•−^, forming peroxynitrite radicals (ONOO^−^) [47], which leads to endothelial nitric oxide synthase (eNOS) uncoupling and further O_2_^•−^ production. The O_2_^•−^ can also participate in the Haber–Weiss reaction, which interacts with H_2_O_2_ to produce HO^−^. [46]. Superoxide dismutase (SOD) is critical for the O_2_^•−^ reduction to H_2_O_2_. The H_2_O_2_ also participates in the Fenton reaction, which reacts with iron ions in their reduced state (Fe^2+^) to generate HO^−^. Xanthine oxidase (XO) and nicotinamide adenine dinucleotide phosphate (NADPH) oxidase (NOX) also produced HO^−^. The HO^−^ is cleared by catalase (CAT), glutathione peroxidase (GPX), and the thioredoxin (TRX) system [46,48,49]. The sources of ROS production are the mitochondrial activity at complexes 1 and 3, NOX, XO [50], and uncoupled eNOS [51,52], as well as the Fenton and Haber–Weiss reactions, which generate hydroxyl radicals and contribute to oxidative damage [53]. Figure 4 summarizes the sources of ROS production and its interaction with the antioxidant system.

At physiological levels, ROS maintains the signaling pathways that control cellular processes. Inflammation, differentiation, proliferation, apoptosis, and the immune response processes are regulated by ROS [54]. Oxidative stress overstimulates the mentioned pathways, promoting ROS-associated processes, such as atherosclerosis development [55]. Of note is the feedback in the elevated ROS production, which even provokes more ROS generation, a phenomenon known as ROS-induced ROS release. This effect produces a repetitive cycle of damage and dysfunction, promoting further disease progression [56]. ROS also promotes a pro-inflammatory response, another central factor in preserving ED, via the activation of the transcription nuclear factor-κB (NF-κB) [13,15]. Furthermore, in endothelial cells, NF-κB promotes the expression of pro-inflammatory cytokines such as IL-6 and tumor necrosis factor-alpha (TNF-α) [57], which induces mitochondrial ROS production by NOX activity [58]. Ox-LDL also promotes a pro-inflammatory phenotype, upregulating the expression of oxidized low-density lipoprotein receptor-1 (LOX-1) in endothelial cells, thus contributing to endothelial inflammation and EA status [59]. Additionally, ROS decreased the production and bioavailability of the NO^•−^ produced by the eNOS or any NOS isoform and by the uncoupling of NOS, generating more ROS [13]. Hence, another critical point that should not be ignored is the vicious circle between oxidative stress and inflammation, contributing to maintaining an ED status.

BP-induced ROS have been related to NF-κB, mitogen-activated protein kinase (MAPK) activation, and phosphatidylinositol 3-kinase (PI3K) downregulation. Evidence shows that BPA, BPAF, and BPF activate c-Jun N-terminal kinase (JNK), extracellular signal-regulated kinases (ERK), and NF-κB, inducing inflammation and apoptosis pathway activation [60,61,62,63]. BPA, BPS, and BPAF also activate the p38 MAPK (p38) pathway [64,65,66]. It has been demonstrated that after acute exposure, BPA, BPS, BPAF, and BPF exposure increase serum malondialdehyde (MDA) levels, an oxidative stress biomarker produced by lipid peroxidation. Table 1 shows evidence from representative studies on BPs’ effects on the oxidant system. 

Other effects related to BPA exposure reduce mitochondrial membrane potential, which affects the mitochondria’s efficiency in producing adenosine triphosphate (ATP), favoring ROS production [67] and driving the cell to release Cytochrome C (Cyt C), which activates the caspase pathway [68]. Also, it has been demonstrated that BPA can indirectly modulate NOX activity through an increase in AngII, which enhances these enzymes’ activity [69]. This NOX activation increase leads to high ROS levels that oxidate tetrahydrobiopterin (BH_4_) and more O_2_^•−^ production by uncoupled eNOS [70]. In human umbilical vein endothelial cells (HUVECs) models revealed that BPA increased NO^•−^ production, mRNA expression, eNOS protein levels [71], and phosphorylation [72]. In HepG2 and HaCaT cell lines, BPA increases eNOS expression and protein levels [73]. Similar findings have been reported in C57BL/6 mice in this setting.

**Figure 5:**
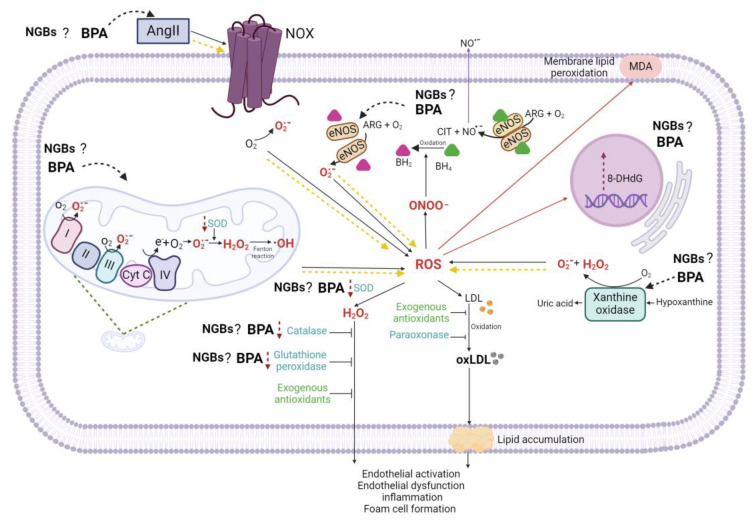
Bisphenol A targets the oxidative and antioxidant systems. Bisphenol A (BPA) indirectly modulates the nicotinamide adenine dinucleotide phosphate (NADPH) oxidase (NOX) activity through increased angiotensin II (AngII), which enhances NOX activation. The increased activated NOX generates more ROS. BPA may also mediate EA by altering the stability of eNOS, promoting the oxidation of BH_4_ to BH_2_ and contributing to further ROS production. Additionally, BPA induces XO, increasing ROS production. BPA significantly reduces the activity of mitochondrial respiratory chain complexes, inducing mitochondrial dysfunction and thus further ROS production. BPA decreases antioxidant enzymes SOD, CAT, and GPX. Overall, BPA exacerbates ROS generation by increasing the activity of oxidative system components (yellow dashed arrows) while reducing the activity of antioxidant enzymes (purple dashed arrows), leading to elevated levels of oxidative stress markers such as malondialdehyde (MDA) and 8-oxo-2′-deoxyguanosine (8-OHdG) (red arrows). Putative NGP targets in the oxidative and antioxidative systems are also indicated. The image was created with BioRender (https://www.biorender.com/ (accessed on 25 September 2024)).

Akin to NOX and XO, BPA and BPs have been linked to ED mediated by the alteration of eNOS [71] and XO induction. BPA 3,4-quinone, an oxidized derivative of BPA, can convert xanthine dehydrogenase into xanthine oxidase, increasing ROS levels [51]. This effect has also been reported for BPB in zebrafish embryos [74]. BPS, BPF, and BPAF in non-nucleated cells enhance ROS production and lipid peroxidation, decreasing some antioxidant enzymes [75]. BPA, BPS, and BPF mainly induce ROS production through NOX expression and activity induction [76,77,78]. In Figure 5, BPs targets in the oxidative system are shown.

**Table 1:** BPA effects on oxidant system parameters related to cardiovascular disease development.

Bisphenol	Study Model	Doses or Level	Total ROS	NO^•−^	MDA	3-NT	eNOS (mRNA or Activity)	8-OHdG	SOD	CAT	GPx	GSH	Mn-SOD	Reference
BPA	HUVECs	10^−6^ M		↑			&↑							[71]
BPS				↑			&↑							
TBBPA				↓			&↓							
TBBPS				↓			&↓							
BPB	Zebrafish	10, 100 and 1000 μg/L	↑		↑				&↓	&↑			*↑	[74]
BPS	HUVECs	25 nM	↑	↓			&↓		&↓		*↓			[79]
BPA	HepG2 cells	50 and 10 μM	↑	↑		↑	*↑						*↑	[73]
	HaCaT cells		↑	↑		-	*↑						*↑	
BPA	Wistar rats	250 mg/Kg	↑		↑					&↓		↓		[80]
BPA	Male Wistar rats	50 mg/kg			↑				&↓	&↓	&↓	&↓		[81]
BPAF	HCMs cells	200 μg/L	↑		↑				$↓	$↓			*↑	[82]
BPA	Erythrocytes	10–500 μg/ml	↑	↑	↑				&↓	&↓	&↓	↓		[75]
BPS			↑	↑	-				-	-	&↓	-		
BPF			↑	↑	↑				&↓	&↓	-	↓		
BPAF			↑	↑	↑				&↓	&↓	-	↓		
BPA	Male Wistar albino rats	50 mg/Kg		↑					&↓	&↓		&↓		[83]
BPA	Male mice	2 mg/Kg			↑				&↓	&↓		↓		[84]
BPA	Male ICR mice	25 mg/kg								&↓	&↓			[85]
BPAP	Humans	BPAP = 0.54 ng/mL						↑						[86]
BPF		BPF = 0.33 ng/mL						↑						
BPA	RAW264.7 cells	0–50 μM	↑	↑										[87]
BPA	Children with ASD	BPA = 8.67						↑						[88]
BPA	Children with ASD	BPA = 1.66 ng/mL						↑						[89]
BPAQ	Primary rat hepatocyte cultures	2–10 μmol/L		↑				↑						[90]

ROS = reactive oxygen species; NO^•−^ = nitric oxide; MDA = malondialdehyde; 3-NT = 3-nitrotyrosine; eNOS = endothelial nitric oxide synthase; 8-OHdG = 8-hydroxy-2′-deoxyguanosine; SOD = superoxide dismutase; Cat = catalase; GPx = glutathione peroxidase; GSH = non-enzymatic antioxidant reduced glutathione; Mn-SOD = manganese superoxide dismutase; ASD: autism spectrum disorder. * mRNA; & Activity; $ mRNA + activity.

### 3.3. Bisphenols and the Vascular Antioxidant System

Cells are equipped with a complex network of ROS-scavenging systems that coordinate to mitigate the ROS effects. The antioxidative system represents a set of enzymes and low-molecular-weight compounds that include SOD, CAT, GPX, the TRX system, paraoxonases (PONs), and mitochondrial uncoupling proteins (UCPs) [91]. Figure 4 summarizes the antioxidant enzymes’ functions. SOD and CAT play a relevant antioxidant role at the VE level [92], SOD scavenges O_2_^•−^ catalyzing its dismutation to H_2_O_2_ and O_2_ [93], and CAT reduces H_2_O_2_ to H_2_O and O_2_ [93].

Concerning BPs’ effects on the antioxidant system, an oral administration of 50 mg/kg of BPA in male rats reduced SOD and CAT levels in hepatic tissue [83]. Similar findings were reported through an intraperitoneal injection of 2 mg/kg of BPA, administered over 4 weeks, where SOD and CAT activity were decreased in pancreas cells [84]. According to these two pieces of evidence, the exposure of male mice to 25 and 50 mg of BPA/kg/day for 5 days significantly decreased CAT activity in the liver [85]. BP exposure decreases CAT and glutathione peroxidase (GSH-Px) expression and activity [68,75,94,95]. Nonetheless, the mechanism has yet to be established. Table 1 provides evidence of BPA effects on oxidant system parameters related to CVD development. About NGBs, scarce information on their impact on the vascular endothelium or CVS is available. However, their structural similarity in chemical functional groups could suggest similar adverse effects [75]. In this setting, BPA, BPS, BPF, and BPAF have shown increased SOD activity [68,75,94,95], inducing toxicity by significantly depleting SOD1 mRNA and protein expression [96,97]. Table 1 shows evidence from representative studies on BPs’ effects on antioxidant system mediators, and Figure 5 shows BPs’ targets on the antioxidant system.

### 3.4. Bisphenols’ Effects on Other Parameters Related to CVD Risk

Other relevant parameters related to CVD development, which are altered by BPA and could be directly or indirectly associated with oxidative stress, will be illustrated with representative evidence. Some of these effects are related to the BP-induced disruption of the hemostatic process, BPs’ endocrine disruptor capability, the impact on the activity of ion channels [98], and, remarkably, BPs’ effect on the inflammatory process.

It has been reported that exposure to BPA at a concentration of 200 nM for 24 h extends the time required to stop bleeding in zebrafish, and BPA and BPS increase the time for fibrin clot formation, suggesting that these BPs may affect blood homeostasis and the extrinsic coagulation pathway [99]. In contrast, BPS at doses of 30, 60, and 120 mg/kg/day for 30 days in adult male rats reduces coagulation time, possibly due to an increase in intrinsic clotting factors, mainly through the formation of fibrin from fibrinogen and a rise in Ca^2+^ ion concentration [100]. Meanwhile, BPAF exhibits a biphasic effect; at low doses (25 μM), it activates platelets and promotes a highly procoagulant response, while at high doses (>50 μM), it induces platelet death by causing oxidative damage through mitochondrial activity, Ca^2+^-mediated calpain activation, lysosomal permeabilization, and lipid peroxidation [101].

On the other hand, regarding the effects of BP exposure on the activity of ion channels, evidence has shown that BPA—at nanomolar and micromolar concentrations—inhibited T-type Ca^2+^ channels and plugged the channel pore in kidney cells (HEK 293) [102]. At a high concentration, 10 mM, BPA has been associated with an increased intracellular activity of voltage-sensitive Ca^2+^/K^+^ channels (Maxi-K) in smooth muscle cells [98]. Furthermore, BPA exposure in wild-type mice has been linked to an increase in AngII, which can uncouple the eNOS and has also been associated with an increase in the expression of Ca^2+^/calmodulin-dependent protein kinase II-α (CaMKII-α), which is responsible for Ca^2+^-related signaling pathways in the cell [69].

NGBs also have been related to effects on other parameters associated with the CVS. BPF (7 ng/mL) acts on Ca^2+^ L-type channels, increasing cytosolic Ca^2+^, generating morphological changes in mitochondria, and decreasing ATP production [103]. BPS also alters intracellular Ca^2+^ through estrogen receptor (ER) β, inducing a rapid phosphorylation of ryanodine and phospholamban receptors [104]. In a zebrafish model, BPB, BPF, and BPAF reduced the mRNA expression of the pore-forming subunit of the voltage-gated L-type Ca^2+^ channel (LTCC), and the mixture of BPA, BPB, BPF, and BPAF negatively regulated the expression of the voltage-dependent Na^+^ channel (scn5lab) and sarco/endoplasmic reticulum Ca^2+^-ATPase (SERCA; atp2a2a). Also in zebrafish, it has been reported that these BPs produced bradycardia in a manner that was not dependent on the ER [105].

Recently, docking studies have shown that BPS and BPF can bind to the thyroid hormone receptor β (THRβ), although the highest binding was demonstrated by BPF [106]. BPB, BPS, BPE, and BPF are capable of binding to and activating ERs and androgenic receptors (ARs) in human adrenal cortico-carcinoma cells, with activity like BPA, except for BPS, which was less estrogenic and antiandrogenic than BPA [107]. BPE, BPC, and BPAP also activate the G-protein-coupled estrogen receptor (GPER) in neuroblast cells (IMR-32) [108]. These findings are relevant because hormonal alterations are relevant to risk factors for CVD development [109].

BPs’ effect on inflammation is highly relevant to EA and ED perpetuation since it positively regulates bidirectional ROS production (described in Section 1). Among the pro-inflammatory effects of BPs, the following stand out: in Wistar rats exposed to BPA (50 μg/kg per 30 days), a relevant low dose to humans, BPA alters the production of inflammatory markers [110] and induces an inflammatory phenotype in murine macrophages [111]. In RAW264.7, a murine macrophages cell line, 50 μM BPA increased the production of cytokines IL-1β, IL-12, TNF-α [87], and IL-6 [110] in a concentration-dependent manner; it also increased the expression of inducible nitric oxide synthase (iNOS), cyclooxygenase-2 (COX-2), and prostaglandin E2 (PGE2), as well as the activation of the MAPK p38, ERK, and Jun N-terminal kinase (JNK). It was also reported that BPA induced the activation of signals transducer and activator of transcription (STAT) 1 (STAT1), STAT3, and NF-kB, which are involved in inflammatory processes [87]. Concerning NGBs, pro-inflammatory effects have been reported with low concentrations of BPS in murine RAW264.7 cells. In this same cell, BPS increased pro-inflammatory markers and activated NLRP3 (NACHT, LRR, and PYD domain-containing protein 3), toll-like receptor 4 (TLR4), and the MAPK pathway [112]. Similar results were induced by BPF in the RAW264.7, increasing the expression of inflammatory mediators through activation of the JAK2/STAT3/ and the suppressor of cytokine signaling 3 (SOCS3) pathways [112]. On the other hand, BPAF has been linked to STAT1 activation [44].

Recently, evidence showed BPs’ impact on neutrophils, suggesting a potential thrombo-embolic promotion by these compounds. BPA at 16 nM to 12 μM induced changes in the immunophenotype of PMN V [113] and 0.03–100 μM in human neutrophils induced ROS production, an effect dependent on Ca^2+^ and ERβ, and reduced its chemotactic capacity [114]. Additionally, BPA altered differentially the neutrophil’s functions depending on the sex, decreasing the phagocytic activity and NETs releasing, inducing NADPH activity, NO production, as well as iNOS and PI3K-Akt expression [115]. In neutrophils from chicken, PBA also inhibited the NETs releasing and activated the p38-MAPK pathway and concomitantly induced oxidative stress [116].

Other central molecules to the ED, ICAM-1 and VCAM-1, are also affected by BPs. The ICAM-1 regulates the leukocyte trafficking and leukocyte trans-endothelial migration; it is expressed at a low basal level in endothelia and is upregulated by pro-inflammatory stimuli [117]. The activated endothelial cells express VCAM-1, which is selectively bound to monocytes, promoting its extravasation. It also has a central role in the genesis of atherosclerotic lesions [118]. Even though evidence on BPs’ effect on ICAM-1 and VCAM-1 is yet limited, in HUVECs, BPA, in addition to induced ROS, increased IL-8, IL-1, monocyte chemotactic protein-1 (MCP-1) and VCAM-1, ICAM-1, and E-selectin [119]. In cumulus cells from infertile women, BPA increased the mRNA expression and protein levels of ICAM-1 and altered DNA methylation profiles [120]. In contrast, in a cell model of first-trimester trophoblast cells, 1 nM BPA downregulated ICAM-1 and altered DNA methylation of the stress response [121].

### 3.5. Association of Bisphenol Exposure to CVDs in Humans

Many of the effects of BPs observed in *in vivo* and *in vitro* models have also been reported in human studies. The levels of BPs have been associated with CVDs such as hypertension, ischemic heart disease, high-triglyceride and high-cholesterol profiles [122], dilated cardiomyopathy, coronary artery disease, congestive heart failure, angina pectoris [28], acute and chronic inflammatory markers, and heart attack or stroke, among others [123]. Multivariate models adjusted for relevant covariates have shown that BPA levels in urine increase CVD risk (OR = 1.09, CI = 1.01–1.18, *p* < 0.05) [124]. Table 2 shows data from representative studies on BPs’ adverse effects on human cardiovascular health.

The contribution of BP exposure has been explored in populations of different ages because aging is a known risk factor associated with the development of CVDs [136]. Exposure to BPs has been shown to increase the risk of CVDs in various age groups. Of note are the effects on 10-year-old children and the relevant maternal urine BPA levels found, which were associated with alterations in the carotid intima–media thickness [137]. In healthy young adults, BPA levels have been strongly associated with increased levels of lipases, short-chain, and unsaturated lipids [138]. In adults, BPF was associated with congestive heart failure, while BPS was positively related to the development of hypertension [29]. In an elderly population, exposure to BPA was associated with a reduction in vitamin D levels, which has been associated with an increased risk of CVDs [139].

Since hypertension is one of the main risk factors for the development of CVDs, the contribution of exposure to BPs has also been evaluated in humans. BPA and BPS increased the risk of hypertension and high systolic blood pressure, with a more prominent association in males than females [131]. On the contrary, another study reported that BPA was associated with a higher prevalence of CVDs in men, BPF was associated with a higher prevalence of CVDs in women, and BPS was not associated with CVDs [134]. Similarly, high levels of BPA in urine have been linked to the incidence of coronary artery disease (CAD) [125]. In recent years, an increased incidence of CAD has been observed in young people [138]. This increase could be related to high levels of BPA in urine, which has been associated with a high body mass index (BMI), blood pressure, plasma levels of C-reactive protein (CRP), and ERβ and Kβ expression [132]. All these factors are well-known comorbidities and risk factors for CVDs. In a population exposed to higher levels of BPA, there exist increased levels of high-sensitivity CPR (hs-CPR), a marker of acute inflammation [129].

## 4. Conclusions

CVDs represent the leading cause of death worldwide. Nowadays, the role of environmental pollutant exposure, such as BP compounds, has gained attention for their effect on this health problem. BPA, the most extensively used BPs in the plastic industry, induces cardiovascular toxicity and is potentially associated with the risk of developing cardiovascular diseases. Based on the evidence of BPA toxicity in different organs and systems, including the CVS, the plastic industry introduced BPA’s structural analogs, arguing that their configuration changes could diminish secondary effects. Nevertheless, NGB toxicity has not been studied yet. The evidence supports that BPA induces a high level of ROS at the endothelial level and consequently induces EA → ED, causing inflammation and promoting a repetitive cycle of ROS production–inflammation, representing a real risk for CVD development.

Despite the scarce evidence on pro-oxidant effects associated with NGBs, our review demonstrated a comparable harmful effect on BPA. Also, we shed light on the cardiotoxicity of NGBs, which exhibited pro-oxidant effects, representing also a real risk to the cardiovascular system. Yet, this review can also be extrapolated to other complex diseases such as cancer and interstitial lung, kidney, and reproductive pathologies, among others. Despite the evidence of NGBs’ effects on the CVS, readers should not lose sight of the fact that CVDs are complex, and family history, genetic burden, and lifestyle factors play a critical role in their development. More studies on NGBs’ effects on the CVS, particularly at molecular mechanistic levels, are still necessary.

In conclusion, NGBs are not a secure alternative to substitute BPA in plastic manufacturing because of their capability to induce oxidative stress generation, which is a risk of inducing atherosclerotic plaque formation and progression. Nevertheless, further studies are necessary to support NGBs’ effects on the oxidant and antioxidant systems using vascular tissue models. Our review opens the door to new studies in different models and populations to contribute to the future directions regarding public policies to regulate bisphenol use, given its impact on health, particularly at the CVS level.

## Figures and Tables

**Table 2:** Representative studies on bisphenols’ adverse effects on human cardiovascular health.

Study Design	Country	Total *n* (*n* with CVDs)	Matrix	Analytical Technique Used	Average Levels of Bisphenol	Estimated Risk	Effect	Adjustment Factor	Reference
Prospective	UK	1619 (758)	Urine	HPLC/MS	BPA 1.3 ng/mL	OR = 1.13, CI = 1.02–1.24	BPA levels were positively associated with the incidence of coronary artery disease in adjusted multiple models.	Age, sex, and urinary creatinine.	[125]
Cross-sectional	USA	753 (63)	Urine	GC/MS	BPA 2.30 ng/mL	OR = 2.69, CI = 1.02–7.09	BPA levels (before and after multivariate adjustment) were positively associated with peripheral arterial disease.	Age, sex, ethnicity, BMI, education, income, smoking status, pack-years of smoking, alcohol intake, hypertension, diabetes, eGFR, urinary creatinine, and total cholesterol.	[126]
Cross-sectional	USA	2200	Urine	HPLC/MS	BPA 4.8 ng/mL	OR = 3.80, CI = 2.25–6.43	Increasing urinary BPA levels were positively associated with obesity, predominantly in non-Hispanic white children.	Age, ethnicity, education, moderate activity, urinary creatinine, and serum cotinine.	[127]
Cross-sectional	Italy	139	Plasma	ELISA	BPA 1.04 ng/mL	β = 0.298, *p* = 0.008, (WC); β = 0.237, *p* = 0.0347 (IL-6).	BPA concentrations were positively correlated with WC, TG, and glucose and inflammatory cytokine levels	WC, HOMA-IR, IL-6, and TNFα.	[128]
Prospective	Republic of Korea	200	Urine	HPLC/MS	BPA 2.057 μg/L	OR = 2.85, CI = 1.16–6.97	BPA levels were positively associated with hs-CPR levels.	Obesity, BMI, insulin resistance, visceral fat volume, adiponectin, HDL, and HbA1c.	[129]
Prospective	China	1872	Urine	LC/MS	BPA 1.14 μg/g creatinine	R = 2.26, CI = 1.54–2.99 (LDL) R = −0.70, CI = −1.10–−1.31 (HDL) R = −1.08, CI = −1.92–−0.23 (TG)	BPA was negatively associated with HDL and TG levels.	Age and sex.	[122]
Cross-sectional	USA	9139	Urine	SPE-HPLC-MS/MS	BPA 0.4 ng/mL	OR = 1.73, CI = 1.11–2.69 (myocardial infarction); OR = 1.61, CI = 1.09–2.36 (stroke)	Higher levels of BPA were associated with a higher prevalence of myocardial infarction and stroke, heart failure, coronary heart disease, angina pectoris, and stroke.	Age, gender, race, BMI, education levels, marital status, smoking, physical activity, energy intake, diabetes, hypertension, and family history of CVD.	[28]
Prospective	USA	3883	Urine	HPLC/MS	BPA: T1 = 0.7 ng/mL T2 = 2.1 ng/mL T3 = 5.7 ng/mL	HR = 1.51, CI = 1.07–2.13 (BPA) OR = 1.40, CI = 1.03–1.91, (BPS) OR = 1.48, CI = 1.072.05	High exposure to BPA was associated with an increased risk of death.	Adjusted for age, sex, ethnicity, and urinary creatinine levels.	[130]
Cross-sectional	China	1437 (433)	Urine	HPLC	BPA 0.71 μg/L BPS 0.33 μg/L (in HBP)		High levels of BPA and BPS were associated with an increased risk of hypertension and systolic blood pressure.	Urinary creatinine, age, sex, BMI, smoking status, exercise frequency, education, income level, hyperlipidemia, and eGFR.	[131]
Cross-sectional	China	90	Plasma	UPLC/MS	BPA 4.34 ng/mL	OR = 2.61, CI = 1.03–10.6	High BPA levels were associated with a higher risk of high BMI, blood pressure, CPR, Erβ and IKKβ levels, and coronary artery disease.	Gender, age, BMI, systolic and diastolic blood pressure, BPA exposure, CRP level, ERβ, and IKKβ.	[132]
Prospective	USA	8164 (740)	Urine	HPLC–MS/MS	BPA 3.38 ng/mL	OR = 1.09, CI = 1.01–1.18	Higher BPA levels had an increased risk of developing cardiovascular disease.	Age, gender, race, education, poverty–income ratio, BMI, drinking, smoking, and activity.	[124]
Cross-sectional	USA	960	Urine	HPLC/MS	BPA 1.1 ng/mL BPS 0.5 ng/mL BPF 0.25 ng/mL	BPA OR = 1.58, CI = 1.10–2.27 BPS OR = 1.80, CI = 1.25–2.60	Highest BPA and BPS levels were associated with the development of non-alcoholic fatty liver disease (NAFLD). No detected association with BPF.	Ethnicity, education, HDL, drinking, hypertension, diabetes, glucose, log-transformed triglyceride levels, glycosylated hemoglobin A1c, urine creatinine, and log-transformed concentration of BPA and BPS.	[133]
Prospective	USA	3502 (368)	Urine	HPLC/MS	BPA 1.20 ng/mL, PBS 0.30 ng/mL, and BPF 0.50 ng/mL (CVD)	BPA OR= 1.58, CI = 1.08–2.30	BPA was associated with a higher prevalence of CVD in men, mainly in non-Hispanic whites, those who were obese, smokers, and with hypertension, diabetes, and a family history of heart attack. BPF was associated with a higher prevalence of CVD in women.	Age, sex, urine creatinine, race, education level, family income, BMI, recreational physical activity, smoking status, drinking status, diabetes, hypertension, and family history of heart attack.	[134]
Prospective	USA	604	Urine	HPLC–MS/MS	BPA 2.5 ng/mL BPF 0.3 ng/mL BPS 0.007 ng/mL	BPA β = 4.015, *p* < 0.05 BPA + BPF β = 1.54, *p* < 0.05	In women, BPA and BPF were associated with a longer PR interval and increased QRS duration.	Not Data Available	[135]

β = beta coefficient; BMI = body mass index; CVD = cardiovascular disease; eGFR = estimated glomerular filtration rate; GC = gas chromatography; HBP = high blood pressure; HbA1c: hemoglobin A1c; HDL = high-density lipoprotein; HPLC = high-performance liquid chromatography; HOMA-IR = homeostatic model assessment for insulin resistance; LC/MS = selective liquid chromatography–tandem mass spectrometry; LDL = low-density lipoprotein; MS = mass spectrometry; SPE = solid-phase extraction; TG = triglycerides; UK: United Kingdom; USA: United States of America; WC = waist circumference.
